# Solitary testicular metastasis post-prostatectomy for prostatic ductal adenocarcinoma: case report and literature review

**DOI:** 10.3389/fonc.2025.1464446

**Published:** 2025-02-27

**Authors:** Bo Chang, Manqing Zhang, Yifan Hou, Wenbin Li, Song Li, Jianhua Zhang, Chenyang Wang, Qiangqiang Zhang, Junqing Hou

**Affiliations:** ^1^ Department of Urology, Huaihe Hospital of Henan University, Kaifeng, Henan, China; ^2^ Department of General Medicine, the First Affiliated Hospital of Henan University, Kaifeng, Henan, China; ^3^ Medical College of Henan University, Kaifeng, Henan, China; ^4^ Department of Urology, Kaifeng 155th Hospital, Kaifeng, Henan, China

**Keywords:** prostatic ductal adenocarcinoma, metastatic testicular tumor, solitary metastasis, laparoscopic radical prostatectomy, orchiectomy

## Abstract

**Background and Purpose:**

Prostatic ductal adenocarcinoma (PDA) constitutes a rare and notably aggressive histological subtype within the spectrum of prostate malignancies, distinguished by a heightened propensity for recurrence and metastasis compared to prostatic acinar adenocarcinoma (PAA). Testicular metastasis in PDA is exceptionally rare. Despite sporadic reports in the literature, a consensus regarding the optimal therapeutic approach remains elusive. This study retrospectively analyzes a singular case of PDA manifesting with solitary testicular metastasis after laparoscopic radical prostatectomy (LRP), consolidating insights into clinical, histopathological, molecular, and therapeutic aspects, alongside existing scholarly discourse.

**Methods:**

We present the case of a 63-year-old gentleman diagnosed with pure PDA (pT3aN0, Gleason score 4 + 4 = 8), exhibiting a serum prostate-specific antigen (PSA) level exceeding 100 ng/ml. Subsequently, the patient underwent androgen deprivation therapy (ADT) followed by LRP. Subsequently, at 17 months post-LRP, local recurrence and a right testicular mass emerged, prompting pelvic radiotherapy and docetaxel chemotherapy. Ultimately, the patient underwent right orchiectomy 65 months post-LRP, with pathological findings confirming metastatic PDA. Four months post-orchiectomy, PSA levels declined to 1.77 ng/ml. Additionally, a comprehensive review of published literature concerning PDA complicated by testicular metastasis was conducted.

**Results:**

The patient derived therapeutic benefits from ADT, LRP, radiation therapy, and orchiectomy, resulting in objective symptom alleviation and a reduction in PSA. Nevertheless, docetaxel proved inefficacious. The literature review indicated variability in outcomes across diverse treatment modalities.

**Conclusions:**

Prolonged surveillance is imperative for patients diagnosed with PDA. Urologists must remain vigilant regarding uncommon sites of metastasis, particularly in instances of elevated PSA.

## Introduction

Prostate cancer (PC) stands as the second most prevalent malignancy among men worldwide (1). Prostatic epithelial tumors exhibit various histological subtypes, including acinar adenocarcinoma (AA), ductal adenocarcinoma (DA), intraductal carcinoma (IDC), squamous cell carcinoma, and basal cell carcinoma. Among these, prostatic ductal adenocarcinoma (PDA) ranks second in incidence, comprising 2.6% of cases (2, 3). PDA is delineated into pure type and mixed type (mixed with AA); pure ductal adenocarcinoma accounts for merely 0.2% to 0.4% of all prostate cancers (2). The description of ductal adenocarcinoma of the prostate dates back to 1967, attributed to Melicow and Pachter (4). Predominant clinical manifestations of PDA include lower urinary tract symptoms and hematuria. Diagnosis of PDA relies on histopathological evaluation, revealing characteristic morphological features such as papillary, cribriform, and solid structures (5, 6).

The long-term prognosis of PDA primarily hinges upon the presence or absence of metastasis, typically affecting bone, lymph nodes, and viscera, while testicular metastasis is a rare occurrence (7). Testicular tumors are predominantly primary germ cell neoplasms, with secondary metastases being exceedingly rare (8, 9). While clinical evidence indicates that metastatic tumors in the testis primarily arise from the prostate, there exists a paucity of robust research and substantial clinical data concerning the metastasis of PDA to the testis (10). This article presents a rare case of PDA with solitary testicular metastasis occurring 17 months post-laparoscopic radical prostatectomy, accompanied by a comprehensive review of published literature detailing the clinical characteristics and treatment strategies for this disease.

## Case report

**Figure 1:**
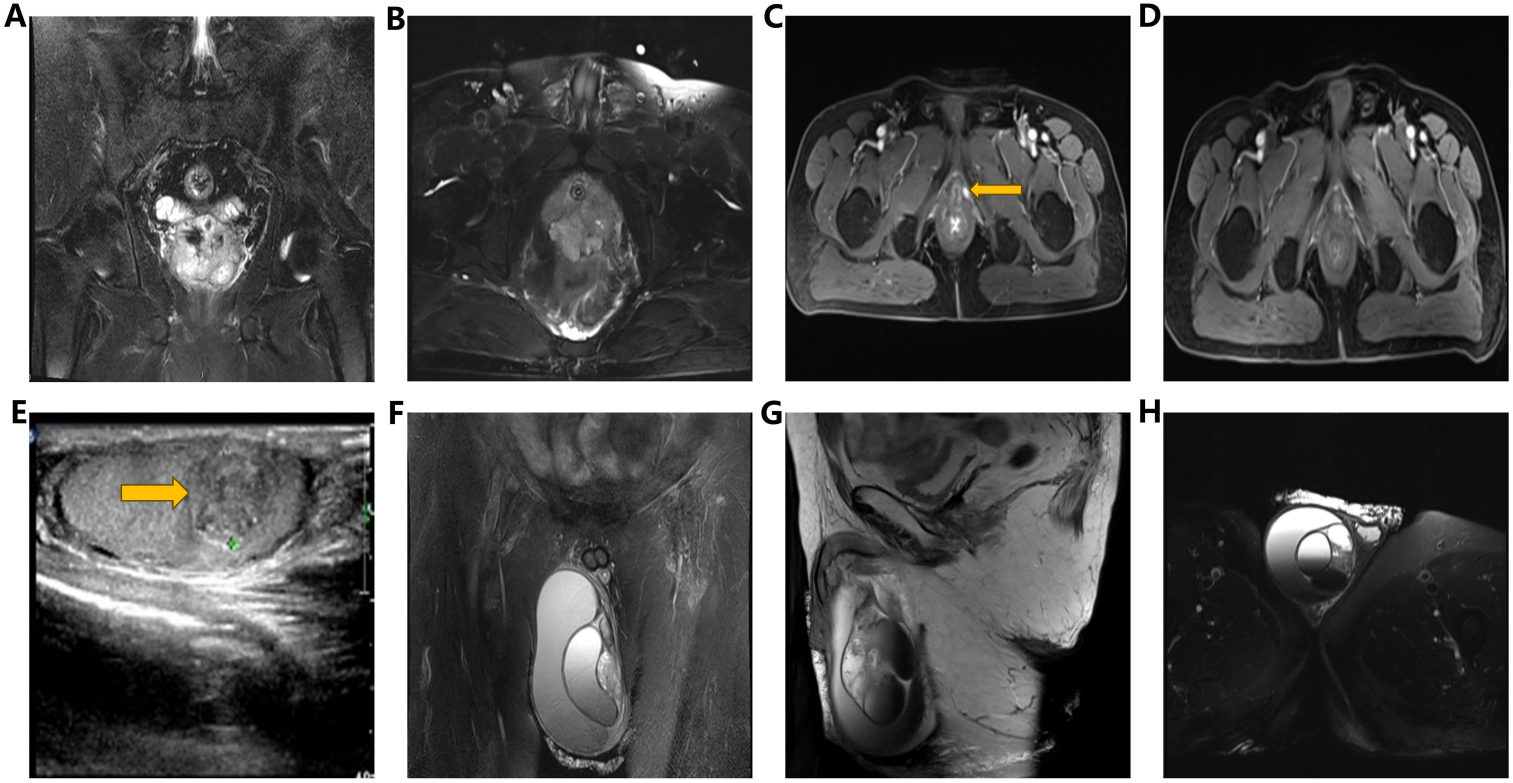
**(A, B)** Pelvic MRI images (coronal view, transverse view): Demonstrating an enlarged prostate volume with irregular shape and indistinct boundary between peripheral zone and urethral periphery zone, along with multiple T2WI low signal nodules and masses, accompanied by focal areas of hemorrhage. **(C)** Pelvic enhanced MRI image (transverse view): Revealing a nodular lesion (1.3cm) on the left perineum, exhibiting significant enhancement upon contrast administration. **(D)** Pelvic enhanced MRI (transverse view): Illustrating resolution of pelvic nodules five months post-radiotherapy. **(E)** Testicular ultrasound: Identifying multiple heterogeneous hypoechoic lesions (1.3×1.0×1.2cm) within the right testicular parenchyma. **(F–H)** Scrotal MRI images (coronal, sagittal, and transverse views): Demonstrating volumetric enlargement of the right testis with multiple intratesticular nodules, accompanied by a large cystic lesion in the right scrotum, suggestive of hemorrhagic changes.

**Figure 2:**
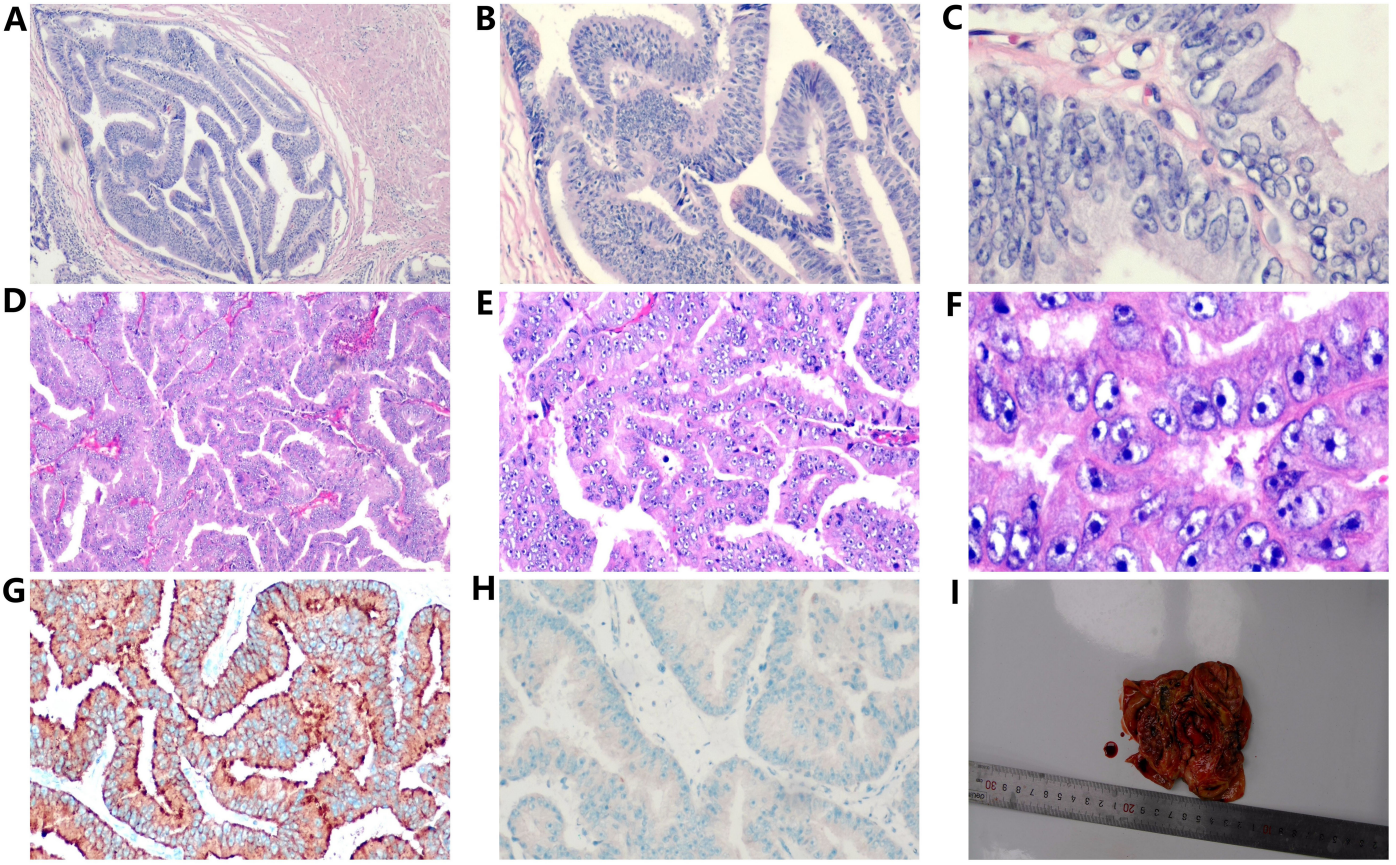
Histopathological examination of prostatic ductal adenocarcinoma **(A–C** and a testicular tumor **(D–F)**, revealing characteristic papillary and cribriform structures with enlarged and hyperchromatic nuclei (H&E, ×40, ×100, ×400). Immunohistochemical results of testicular tumors, **(G)** positive prostate specific antigen (PSA) in the cytoplasm (×100). **(H)** P504S(negative, ×100). **(I)** The gross specimen of the right testis exhibited a gray-white nodule (2.7×2.5×1.5cm) surrounded by cystic fibrous tissue upon sectioning.

In March 2018, a 63-year-old male patient was admitted with a chief complaint of intermittent dysuria and initial hematuria for two years. His medical history included 20 years of bronchitis and 10 years of hypertension. Upon admission, the patient declined a digital rectal examination. Serum prostate-specific antigen (PSA) was found to be >100 ng/mL. Color Doppler ultrasound revealed a residual urine volume of 1100 mL, and pelvic magnetic resonance imaging (MRI) indicated a prostatic mass with minor hemorrhagic foci, suggestive of prostate cancer (**Figures 1A, B**). Chest and abdominal computed tomography (CT) scans, as well as a whole-body bone scan, showed no evidence of distant metastasis. Transrectal prostate biopsy confirmed PDA, characterized by a Gleason score of 4 + 4 = 8. Immunohistochemistry demonstrated positivity for P504S and PSA, with a Ki-67 index of 20%, while 34BE12 and P63 were negative. Following comprehensive discussions with the patient regarding treatment options, including their benefits and anticipated outcomes, the patient opted for neoadjuvant endocrine therapy to reduce tumor volume, enhance local control, and reduce the likelihood of positive surgical margins. Subsequently, the patient underwent a planned 12-week course of androgen deprivation therapy (ADT) to maximize androgen blockade, utilizing a goserelin acetate sustained-release implant (10.8 mg every 12 weeks) and bicalutamide (50 mg daily). This constituted the preoperative neoadjuvant endocrine therapy. However, one month later, the patient was readmitted due to intolerable discomfort from the indwelling urinary catheter. At this time, his serum PSA level was 36.36 ng/mL. At his request, a laparoscopic radical prostatectomy (LRP) was performed. Postoperative pathological examination revealed PDA staged as pT3aN0, with a Gleason score of 4 + 4 = 8. The tumor exhibited diffuse invasive growth, extensively infiltrating the left and right lobes, middle and posterior lobes, constituting approximately 80% of the tissue. Local invasion of the urethral mucosa and prostate capsule, with no cancer involvement observed in the bilateral seminal vesicle glands, vas deferens, or at the upper and lower urethral incisal margins (**Figures 2A–C**).

Seventeen months post-LRP, the serum PSA level measured 16.50 ng/ml, while pelvic MRI revealed a dubious nodule in the left perineal region (**Figure 1C**). Color ultrasonography detected a solid mass within the right testicular parenchyma (**Figure 1E**). Testicular tumor markers indicated human chorionic gonadotropin at 3.73 mIU/ml, while lactate dehydrogenase, alpha-fetoprotein, and carcinoembryonic antigen levels remained within normal limits. We recommended the patient undergo testicular biopsy or orchiectomy. However, due to economic constraints, the patient declined. Subsequently, the patient underwent pelvic radiotherapy (72.9Gy/27f) in conjunction with ADT (goserelin acetate plus bicalutamide). Five months post-pelvic radiotherapy, reassessment revealed a serum PSA level of 4.03 ng/ml, accompanied by the resolution of suspicious nodules on the left perineal side as evidenced by pelvic MRI (**Figure 1D**).

**Figure 3:**
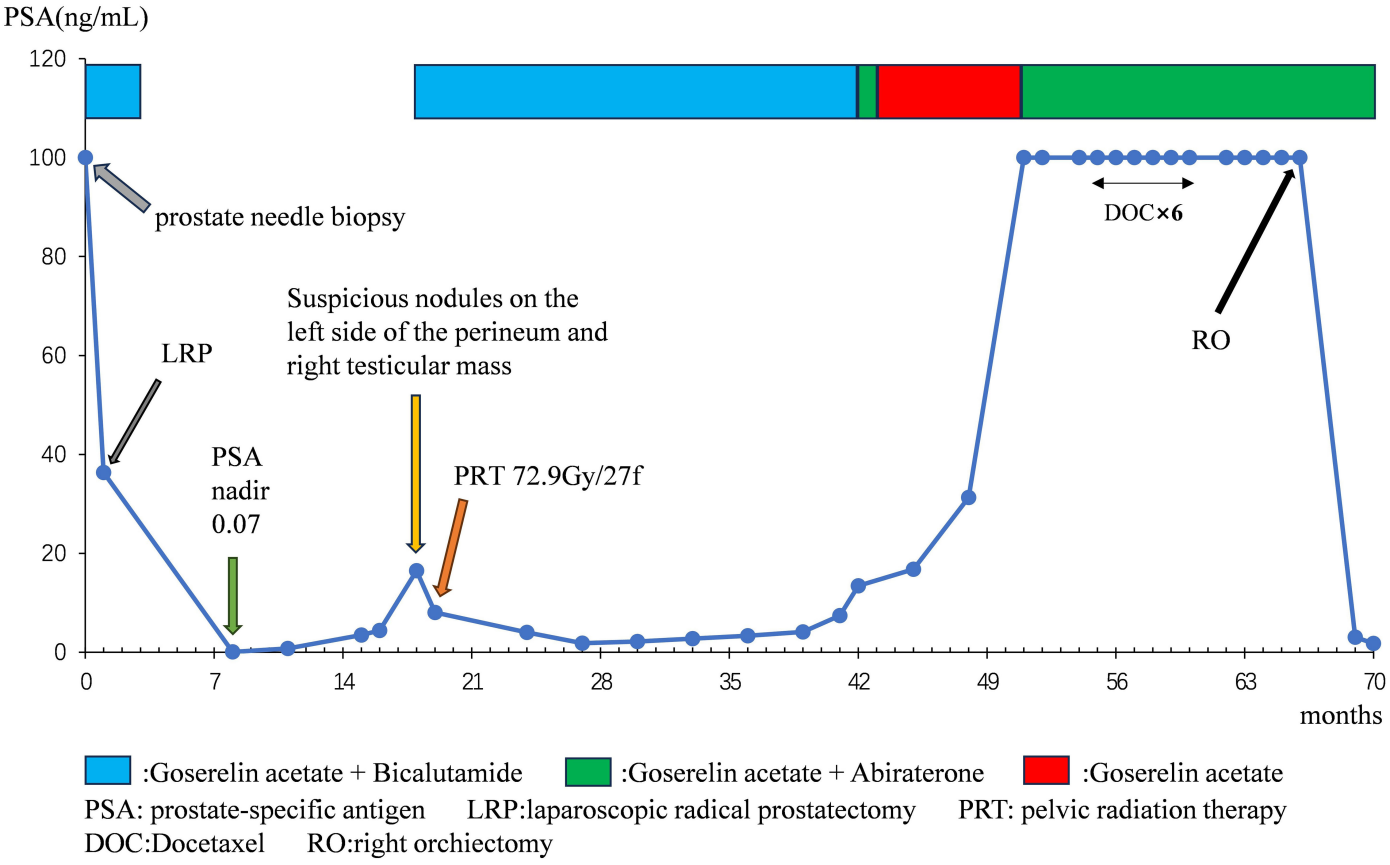
PSA change curves throughout treatment.

At 41 months post-LRP, the serum PSA level measured 13.43 ng/ml, prompting a transition from bicalutamide to abiraterone (1g, once daily). However, after one month of treatment, the patient independently ceased the medication. By 54 months post-LRP, the serum PSA level exceeded 100ng/ml, leading to the initiation of oral abiraterone and docetaxel (75mg/m2, every 4 weeks) chemotherapy. Despite completing 6 cycles of chemotherapy, the serum PSA level remained elevated (> 100ng/ml), and the painless swelling of the right scrotum worsened. At 65 months post-LRP, the patient experienced intolerable pain in the right testicle, with a serum PSA level >100 ng/mL. Scrotal MRI revealed enlargement of the right testis with multiple nodules, along with a large cyst-like structure in the right scrotum indicative of hemorrhagic changes (**Figures 1F-H**). Extensive investigations found no evidence of distant metastasis, and testicular tumor markers remained within the normal range. Consequently, a right orchiectomy was performed, unveiling metastatic PDA with a 2.7×2.5×1.5cm tumor invading the rete testis. No carcinoma was detected at the spermatic cord stump. Immunohistochemistry indicated negativity for P504S and positivity for PSA, with a Ki67 index of 10% (**Figures 2D-I**). In the third month post-orchiectomy, the patient’s PSA level decreased to 3.066 ng/ml. In the fourth month, the PSA level further declined to 1.77 ng/ml (**Figure 3**). The patient presently maintains good physical condition and leads a normative lifestyle.

## Literature review

To explore the rarity of this case, we utilized specific keywords in PubMed, Web of Science, and Google Scholar databases. The search terms included “Prostatic ductal adenocarcinoma,” “Ductal adenocarcinoma of the prostate,” “Testicular metastasis,” “Prostatic carcinoma with testicular metastases,” “Prostate cancer metastasis to testis,” and “Prostate cancer spread to testis.” The search was conducted up to April 2024, excluding duplicate articles and reviews. After meticulous screening, a total of three articles were included: one case series and two case reports. Merely six cases of PDA with metastasis to the testis, including this instance, have been elucidated in the literature (11–13). As delineated in **Table 1**.

**Table 1:** Profiles of 6 cases of PDA with testicular metastasis in contemporary literature.

Author/year of publication	Number of cases	Age/years	Side	Gleason score	Time from PDA diagnosis to testicular metastasis	PSA at Testicular Metastasis (ng/ml)	Site of metastasis	Treatment	Outcomes
Tu et al., 2002 (11)	3	45	Unreported	9	Unreported	<4	Testis, bone, LN	ADT, Orchiectomy, Estramustine/docetaxel	The survival time was 243 months
		59	Unreported	9	Unreported	≥4	Testis, bone, lung, bladder, adrenal gland	ADT, TURP, Ketoconazole/doxorubicin and estramustine/vinblastine	The survival time was 181 months
		68	Unreported	8	Unreported	<4	Testis, bone, bladder, gluteal muscle, LN	Testis, bone, bladder, gluteal muscle, LN	The survival time was 34 months
Anila et al., 2012 (12)	1	68	Left	Unreported	4 years	75	Testis, bone	TURP,RT, ADT, Orchiectomy	Multiple bone metastases were treated with palliative care
Mortensen et al., 2014 (13)	1	89	Left	8 (4 + 4)	4 years	41.6	Testis, bone	ADT, Orchiectomy	Unreported
Our csae (2024)	1	69	Right	8 (4 + 4)	17 months	>100	Testis	ADT, LRP, RT, docetaxel, Orchiectomy	There were no signs of disease after orchiectomy

PDA, prostatic ductal adenocarcinoma; PSA, prostate-specific antigen; LN, lymph node; ADT, androgen deprivation therapy; TURP, transurethral resection of the prostate; RT, radiotherapy; LRP, laparoscopic radical prostatectomy.

The average age was 66 years, with a median age of 68 years. The predominant age of onset ranged from 65 to 70 years (50%), while occurrences at the age of 45 were rare. Among these cases, 2 were located on the left side, 1 on the right side, and 3 lacked side-specific reporting. Gleason scores were 9 in 2 cases, 8 in 3 cases, and not reported in 1 case. The time interval from the initial diagnosis of PDA to testicular metastasis was 4 years in 2 cases, 17 months in 1 case, and undisclosed in 3 cases. PSA levels were < 4 ng/ml in 2 patients and ≥4 ng/ml in 4 patients (with levels exceeding 100 ng/ml in this instance). Among the reported cases, 5 exhibited bone metastases, 2 bladder metastases, and 2 lymph node metastases. Treatment modalities included ADT for all patients, orchiectomy in 5 cases, docetaxel chemotherapy in 2 cases, transurethral resection of the prostate in 2 cases, radiotherapy in 2 cases, and LRP in 1 case. Two patients achieved prolonged survival, spanning 243 and 181 months, respectively, while one survived for 34 months, one received palliative care, and one lacked reported outcomes. None of the previously documented patients underwent radical prostatectomy, and all had bone metastases, with three experiencing at least three metastatic sites. The solitary testicular metastasis observed in the present case, following LRP, represents the first reported instance.

## Histopathological characteristics

The standard of excellence for diagnosing PDA is histopathological analysis. Initially denoted as “prostatic endometrioid adenocarcinoma” due to its distinctive histological traits reminiscent of endometrial cancer in females, it is now established that PDA originates from the major prostatic ducts and their secondary counterparts (5, 14). Previously encompassing papillary, cribriform, solid, and PIN-like carcinoma morphologies, the recent WHO classification of prostate cancer redefines PIN-like carcinoma as a subtype of AA (2).

The papillary structure stands out as the hallmark for diagnosing PDA, typified by tall columnar tumor cells arranged pseudostratified amidst fibrovascular bundles. These cells exhibit conspicuous nuclear atypia, elongated oval or ovoid shapes, prominent nucleoli, frequent mitotic figures, and typically dichromatic abundant cytoplasm. Conversely, the cribriform structure, comprising columnar epithelial cells, features slit-like lumens distinct from the round lumens in acinar prostate cancer. Solid structure, albeit rare in PDA, manifests as densely packed columnar epithelial cells in solid formations. It is generally posited that papillary structures predominate in the larger ducts encircling the urethra, while cribriform structures prevail in the smaller surrounding ducts; however, these two morphologies are often intertwined. A Gleason score of 4 + 4 = 8 is designated in the presence of papillary or/and cribriform structures. Immunohistochemical profiles of PDA closely mirror those of AA, frequently exhibiting positive expression of prostate tissue-specific markers (prostate-specific antigen and prostate-specific acid phosphatase). The Ki-67 proliferation index of PDA surpasses that of AA, indicating its heightened biological aggressiveness.

## Discussion

The median age of onset among individuals diagnosed with PDA spans from 60 to 80 years, marginally surpassing that of those afflicted with AA (15). Originating from the periurethral prostatic ducts, PDA manifests chiefly through lower urinary tract symptoms (such as urinary tract obstruction, diminished urine flow, or urinary retention) and hematuria (either microscopic or gross). Hematospermia may ensue upon tumor infiltration into the seminal vesicle glands or urethra (16). Owing to the nonspecific symptomatology, benign prostatic hyperplasia (BPH) often emerges as the initial clinical diagnosis for such cases. The patient in question endured dysuria and initial hematuria persisting for over two years, concomitant with a residual urine volume of 1100ml upon initial assessment. The distinctive growth pattern of tumor cells in PDA culminates in elevated luminal PSA secretion juxtaposed with diminished serum PSA secretion. Consequently, patients with this subtype are 2.4 times more prone to exhibit PSA levels below 4.0 ng/ml compared to those with AA, with a substantial proportion of PDA patients registering PSA levels below this threshold upon diagnosis (17). The PSA level of the patient surpassed 100 ng/ml during the initial hospital visit and fluctuated throughout subsequent treatment. Fifty months post-LRP, the PSA level once again exceeded 100 ng/ml. However, the alteration in PSA sensitivity enables us to more accurately gauge disease progression. Four months after the orchiectomy, the PSA level dropped to 1.77 ng/ml. Despite this significant reduction from over 100 ng/ml to 1.77 ng/ml indicating the efficacy of the orchiectomy, the PSA level did not fall below 0.2 ng/ml. Possible reasons include: first, the presence of a small number of prostate cancer cells that continue to secrete PSA; second, PDA is an aggressive subtype of prostate cancer that often exhibits partial resistance to conventional castration therapy, which may limit the extent of PSA reduction post-treatment. Therefore, we have devised the following clinical management measures for this patient: first, continue regular monitoring of PSA levels and other imaging examinations to assess tumor progression or residual disease; second, consider further treatment based on the patient’s overall condition and response to therapy; third, conduct multidisciplinary team discussions as needed to develop a personalized treatment plan to optimally control the condition.

PDA exhibits greater aggressiveness compared to prostatic acinar adenocarcinoma (PAA). At the time of diagnosis, PDA is three times more prone to metastasize than AA and demonstrates a predilection for metastasizing to uncommon sites such as the brain, skin, penis, and testes (5). Testicular metastasis from any primary site is uncommon, with the testes often termed a “tumor sanctuary” due to the presence of the blood-testis barrier and the unfavorable low temperature of the scrotum for metastatic tumor survival (18, 19). Currently, most instances of prostate cancer with testicular metastasis are documented through autopsy or examination of testicular castration specimens (20). Given that PDA originates from the periurethral prostatic ducts, there exists an elevated risk of prostatic urethral invasion by tumor cells, potentially leading to testicular dissemination via arterial embolization, retrograde venous spread, retrograde lymphatic metastasis, or direct metastasis through the vas deferens (12). In this particular case, no cancer cells were detected in the vas deferens, indicating a likelihood of venous or lymphatic metastasis.

A consensus regarding the optimal management of PDA remains elusive, with treatment modalities resembling those utilized for AA. Radical prostatectomy or radiotherapy represents the primary selection for local intervention, while ADT or chemotherapy constitutes the principal option for systemic management (21). Superior survival outcomes are associated with radical prostatectomy (RP) (22, 23). However, PDA is correlated with an increased propensity for local recurrence following radical prostatectomy. Following RP, patients exhibiting inadequate PSA control, unfavorable pathological characteristics, or a high risk of recurrence may be candidates for postoperative adjuvant radiotherapy. In this instance, the patient underwent radiotherapy 18 months following LRP, resulting in the disappearance of a suspicious nodule in the left perineum and a decrease in PSA levels, thereby validating the efficacy of radiotherapy. Several studies have documented the effectiveness of docetaxel in managing metastatic PDA (11, 24, 25). Nonetheless, the administration of docetaxel chemotherapy in this patient ceased due to exacerbation of scrotal enlargement following six cycles of treatment. A study involving 228 patients with PDA revealed that ADT was less effective in preventing recurrence compared to AA, with over 85% of patients progressing following initial treatment (26). Nonetheless, a similar high expression of androgen receptor was demonstrated in both PDA and AA (27, 28). Given the patient’s rapid PSA elevation following discontinuation of abiraterone after a month of goserelin acetate plus abiraterone, incorporating abiraterone in addition to ADT could be beneficial for patients with PDA.

The incidence of metastatic testicular tumors ranges from 0.02% to 2.5%, with the prostate being the most prevalent primary site, constituting approximately 15% (29). In cases where a patient with PDA presents with a testicular mass and elevated PSA levels, the possibility of testicular metastases should be considered, even post-prostatectomy. Solitary testicular metastases warrant prompt orchiectomy, a simple and efficacious procedure that diminishes the tumor burden in patients. In this particular case, due to financial constraints, the patient was unable to actively engage in treatment. The interval from the discovery of an asymptomatic testicular mass to the eventual orchiectomy, prompted by pain from an enlarged testicular metastasis, was 48 months. Based on these findings, orchiectomy is most optimally performed upon initial identification of the testicular mass.

PDA exhibits a graver prognosis compared to AA (30). The 5-year overall survival (OS) rate for metastatic PDA ranges from 19.4% to 72%, with a median survival time of 77 months. Despite undergoing multiple systemic treatments, 87% of men with newly diagnosed metastases still experience progression (7). Wu et al. analyzed 511 PDA cases from the SEER database, finding that T3 and T4 stages were significantly more common in PDA patients than in AA patients (22.5% vs. 15.8%, respectively). The 5-year cancer-specific survival rate for PDA increased to 72%, yet it remains 20% lower than that for AA (31). In another study involving 581 PDA patients, PDA demonstrated higher aggressiveness and poorer prognosis compared to AA, regardless of the stage (32).Testicular metastasis signifies an advanced stage of PDA, often accompanied by widespread metastases throughout the body. However, the prognosis of PDA with solitary testicular metastasis remains unreported. Studies indicate that many patients with metastases in rare sites experience stable disease, maintain relatively good quality of life, and remain asymptomatic, except for urinary system-related symptoms, before the progression of bone and visceral metastases (11). In this instance, rapid PSA reduction followed testicular resection, yielding apparent clinical benefits. Nonetheless, owing to the highly aggressive nature of PDA, the long-term prognosis remains uncertain, necessitating diligent monitoring.

It is noteworthy that while PSA presently serves as the sole biomarker for prostate cancer, a retrospective analysis of the existing literature indicates that PSA levels may not consistently correlate with the severity of PDA. Therefore, PSA may not be a reliable indicator for evaluating the treatment effect and prognosis of PDA and should be evaluated in combination with clinical symptoms and imaging findings. Compared with conventional imaging, prostate-specific membrane antigen-positron emission tomography/computed tomography (PSMA-PET/CT) can detect metastatic prostate cancer in some uncommon sites with better results than CT, bone scans, and MRI, especially at lower PSA levels (33). However, recent studies have unearthed unsatisfactory results regarding the effectiveness of PSMA-PET/CT in the context of PDA (34–36). One study revealed that this discrepancy stemmed from significantly lower PSMA expression in PDA compared to AA. Additionally, the study noted higher GLUT1 expression in PDA, particularly in membrane GLUT1 expression. Targeting membrane GLUT1 may emerge as a novel anticancer strategy for PDA in the future (37). Furthermore, the high cost of utilizing PSMA-PET/CT must be taken into consideration. Consequently, novel biomarkers are imperative for assessing PDA in the future.

The genomic and transcriptomic characteristics of PDA are similar to AA, but several pathways driving the aggressive phenotype are upregulated in PDA. DNA damage repair (DDR) alterations are more prevalent in metastatic PCa compared to localized PCa (38). DDR alterations are also frequently observed in ductal adenocarcinoma patients. Reports indicate these alterations occur not only in somatic mutations (20%–49%) but also in autosomal dominant germline mutations (20%), with a higher incidence than in metastatic castration-resistant prostate cancer (mCRPC) (39). Thus, germline testing for PDA is recommended to identify therapeutic targets. A study of 51 PDA patients found that DDR gene mutation rates in PDA were higher than in other tumors, twice that of mCRPC (40–42). Compared to AA, mismatch repair (MMR) defects are more common in PDA. In a study of 10 PDA patients, 4 (40%) exhibited MMR gene alterations, with 3 (75%) showing high mutation rates (43). Given the high incidence of MMR defects, molecular profiling, including immunohistochemistry (IHC) for MMR proteins, holds significant predictive relevance. Precision oncology necessitates treatment decisions based on the tumor’s molecular characteristics. In this case of solitary testicular metastasis post-LRP, comprehensive molecular analysis could elucidate the tumor’s genetic landscape. MMR defects, indicated by the loss of expression of proteins such as MLH1, MSH2, MSH6, and PMS2, suggest a high microsatellite instability (MSI-H) status, known to correlate with better responses to immunotherapy, particularly PD-1/PD-L1 inhibitors (44). This molecular characterization aids in understanding tumor biology and potential progression pathways, and is crucial for targeted therapy. IHC detection of MMR protein expression can guide personalized treatment, potentially improving the prognosis and management of metastatic PDA. Therefore, incorporating routine molecular profiling in the diagnostic evaluation of PDA patients is recommended to enhance predictive and therapeutic precision.

## Conclusion

Due to the rarity of PDA, establishing large-scale prospective studies poses a challenge. Present investigations predominantly rely on limited case series and individual case reports. In this study, we present the inaugural case of pure PDA with solitary testicular metastasis after LRP. We aim to synthesize pertinent literature, providing valuable insights for the diagnosis and management of this condition. However, this study has certain limitations. For instance, the patient’s financial constraints precluded the use of advanced imaging modalities such as PET-CT, which may have enhanced the precision of staging and the detection of occult metastases. Accurate diagnosis stands paramount for effective disease management. Healthcare practitioners must heighten their awareness of this condition. Even post-prostatectomy, patients diagnosed with PDA necessitate comprehensive evaluation. This evaluation may include testicular assessment when clinically indicated, particularly in cases with suspected metastasis or unusual clinical presentations. To avoid unnecessary treatment, it is essential to accurately diagnose testicular metastases. In cases where testicular metastases are confirmed, timely orchiectomy should be considered to manage symptoms and prevent complications.

## Data Availability

The original contributions presented in the study are included in the article/supplementary material. Further inquiries can be directed to the corresponding author.
